# Curcumin: Modulator of Key Molecular Signaling Pathways in Hormone-Independent Breast Cancer

**DOI:** 10.3390/cancers13143427

**Published:** 2021-07-08

**Authors:** Reyhaneh Farghadani, Rakesh Naidu

**Affiliations:** Jeffrey Cheah School of Medicine and Health Sciences, Monash University Malaysia, Jalan Lagoon Selatan, Bandar Sunway 47500, Selangor, Malaysia

**Keywords:** triple negative, HER2, hormone-independent, breast cancer, curcumin, signaling pathway, clinical trial, polyphenol, phytochemical, chemotherapy and chemoprevention

## Abstract

**Simple Summary:**

Breast cancer remains the most commonly diagnosed cancer and the leading cause of cancer death among females worldwide. It is a highly heterogeneous disease, classified according to hormone and growth factor receptor expression. Patients with triple negative breast cancer (TNBC) (estrogen receptor-negative/progesterone receptor-negative/human epidermal growth factor receptor (HER2)-negative) and hormone-independent HER2 overexpressing subtypes still represent highly aggressive behavior, metastasis, poor prognosis, and drug resistance. Thus, new alternative anticancer agents based on the use of natural products have been receiving enormous attention. In this regard, curcumin is a promising lead in cancer drug discovery due its ability to modulate a diverse range of molecular targets and signaling pathways. The current review has emphasized the underlying mechanism of curcumin anticancer action mediated through the modulation of PI3K/Akt/mTOR, JAK/STAT, MAPK, NF-ĸB, p53, Wnt/β-catenin, apoptosis, and cell cycle pathways in hormone-independent breast cancer, providing frameworks for future studies and insights to improve its efficiency in clinical practice.

**Abstract:**

Breast cancer is the most frequently diagnosed cancer and the leading cause of cancer death among women worldwide. Despite the overall successes in breast cancer therapy, hormone-independent HER2 negative breast cancer, also known as triple negative breast cancer (TNBC), lacking estrogens and progesterone receptors and with an excessive expression of human epidermal growth factor receptor 2 (HER2), along with the hormone-independent HER2 positive subtype, still remain major challenges in breast cancer treatment. Due to their poor prognoses, aggressive phenotype, and highly metastasis features, new alternative therapies have become an urgent clinical need. One of the most noteworthy phytochemicals, curcumin, has attracted enormous attention as a promising drug candidate in breast cancer prevention and treatment due to its multi-targeting effect. Curcumin interrupts major stages of tumorigenesis including cell proliferation, survival, angiogenesis, and metastasis in hormone-independent breast cancer through the modulation of multiple signaling pathways. The current review has highlighted the anticancer activity of curcumin in hormone-independent breast cancer via focusing on its impact on key signaling pathways including the PI3K/Akt/mTOR pathway, JAK/STAT pathway, MAPK pathway, NF-ĸB pathway, p53 pathway, and Wnt/β-catenin, as well as apoptotic and cell cycle pathways. Besides, its therapeutic implications in clinical trials are here presented.

## 1. Introduction

Cancer as the complex disease is a major cause of morbidity and mortality around the world, with 9.9 million deaths in 2020, and has been considered as the world’s biggest killer by the age of 70 years in most countries in 2019. The world cancer burden is still increasing, with an estimated occurrence of 28.4 million new cancer cases in 2040, which is an increase of about 47% compared to 2020 globally [1,2]. Among the various types of cancer, breast cancer remained the most frequently occurring cancer among women worldwide, with 2,261,419 newly diagnosed cases in 2020, accounting for 1 in 4 cancer cases, and has now surpassed lung cancer in global cancer incidence. Besides, female breast cancer ranks first in terms of mortality, with 684,996 deaths in 2020 accounting for 1 in 6 cancer deaths globally [2]. Although the incidence rates of breast cancer vary worldwide and remain much higher in Australia and New Zealand (95.5 per 100,000), rapid rises have been observed in Africa and Asia. There has been a historically low incidence in Asian countries such as Japan and Korea in recent years [3,4,5].

Breast cancer is well known as a genetically and clinically heterogeneous disorder encompassing numerous subtypes, with distinct histopathological patterns and molecular characteristics resulting in various responses to therapies and clinical outcomes [6,7]. In this respect, the differential expression of the growth factor and hormonal receptors (HR) including the presence or absence of the progesterone receptor (PR) and estrogen receptor (ER), or the amplification/overexpression of the human epidermal growth factor receptor-2 oncogene (HER2), have been considered as the chief determinants of these subtypes [8,9]. 

Approximately, two-thirds of breast cancers are ER+ and/or PR+, which are hormone-sensitive and responsive to endocrine therapy with, aromatase inhibitors and selective ER modulators [10,11]. However, the overexpression of HER2, a tyrosine kinase receptor mediating cell proliferation and survival, occurs in about 15–30% of breast cancer. HER2-positivity has been more frequently reported in HR- compared to HR+ cancers correlated with an aggressive disorder and poor prognosis [12,13,14]. Although HER2-targeted therapies have dramatically improved the overall survival among hormone-independent HER2+ patients, drugs-related side effects are yet major obstacles ahead [12,15,16]. Besides, triple negative breast cancer (TNBC) represents a specific subtype accounting for approximately 15–20% of breast cancers, which is clinically negative for the expression of ER and PR, and lacks HER2 overexpression (ER-, PR-, HER2-). TNBC has a highly aggressive clinical behavior, prone to earlier relapses and often metastasis to the brain and lungs correlated with poorer overall survival compared with other subtypes. This subgroup is also difficult to treat and fails to respond to hormonal therapies or those targeting the HER2 receptors [17,18,19]. 

Therefore, despite the overall successes in breast cancer therapy, challenges to managing and treating hormone-independent breast cancers (HER2+ or HER2-) still remain. Hence, in addition to the conventional medicine, it is urgent to develop more effective agent without side effects. In this regard, chemical entities present in plants are now becoming a significant option in cancer drug discovery. One of the most noteworthy of phytochemicals, curcumin, has attracted enormous attention as a promising drug candidate in breast cancer prevention and treatment [20,21,22,23]. 

Curcumin is the major bioactive constituent of the turmeric spice derived from the rhizome of the plant Curcuma longa L. It has been widely used in traditional Indian medicine (Ayurveda) for the treatment of a variety of diseases for at least 4000 years [24,25]. This golden spice has also been found to exert preventive and therapeutic effects in breast cancer. The preclinical models have demonstrated the pivotal role of curcumin in breast cancer progression through regulating cell survival, proliferation, apoptosis, invasion, angiogenesis, and metastasis [26,27,28,29]. Given to its significance, the current review has highlighted diverse underlying mechanisms of anticancer activity of curcumin in hormone-independent breast cancer mediated via its interaction with numerous signaling pathways. The key intracellular signaling networks are the PI3K/Akt/mTOR pathway, JAK/STAT pathway, MAPK pathway, NF-ĸB pathway, p53 pathway, and Wnt/β-catenin pathway, as well as apoptotic and proliferation pathways. Besides, its therapeutic implications in clinical trials are here presented. In the current review, MDA-MB-231, MDA-MB-435, MDA-MB-436, MDA-MB-468, SUM159, HCC1806, HCC1937, Hs578T, EMT6 and 4T1 cell lines are categorized as hormone-independent HER2 negative breast cancer subtypes, also known as TNBC (ER-, PR-, HER2-), while SKBR-3 and MDA-MB-453 are categorized as hormone-independent HER2 positive breast cancer (ER-, PR-, HER2+) subtypes.

## 2. Curcumin: Impacts on Multiple Cellular Signaling Pathways in Hormone-Independent Breast Cancer

Cell signal transductions are the central processes playing a critical role in cancer progression and development. Existing evidence strongly implies that curcumin interrupts major stages of tumorigenesis, including cell proliferation, survival, angiogenesis, and metastasis in hormone-independent breast cancer through its modulatory effect on the functions of multiple signaling pathways discussed below.

### 2.1. The PI3K/Akt/mTOR Pathway

The phosphatidylinositol-3-kinase (PI3K)/the protein kinase B (PKB or AKT)/the mammalian target of the rapamycin (mTOR) pathway (PAM pathway) involves an intricate signaling cascade linking receptor tyrosine kinase (RTK) to the regulation of cell growth and survival, as well as angiogenesis and metabolism. It is activated by the stimulation of RTK followed by PI3k recruitment and its phosphorylation. After being activated, PI3k triggers the activation of a key signaling kinase AKT, which in turn regulates several downstream effector molecules like mTOR, promoting protein and lipid synthesis and ultimately cell growth. In addition, the activated AKT may inhibit apoptosis and promotes cell survival via the subsequent modulation of various target molecules such as the Bcl-2 family of proteins [30,31,32].

The PAM pathway is the most frequently altered pathway in breast cancer, approximately 70% of cases, and is often activated in the TNBC involved in chemoresistance and survival [32,33,34,35]. Besides, the constitutive activation of the PAM pathway is a potential mechanism of resistance to anti-HER2 therapies [36]. Its oncogenic activation may occur through various mechanisms. The overexpression of the upstream regulator epidermal growth factor receptor, the loss of negative regulators such as proline-rich inositol polyphosphatase and phosphatase and tensin homolog (PTEN), in addition to mutations of the PI3K gene, may result in an upregulated PAM signaling pathway in HR- breast cancer [37,38,39]. Activating the PIK3CA gene mutation is the most common alteration in breast cancer with different frequency among various subtypes identified more than 70% in luminal tumors, 39% in hormone-independent HER2+, and approximately 9% in TNBC [32,40]. However, in TNBC, the loss of PTEN or INPP4B mainly contributes to the dysfunction of the PAM pathway correlated with increased levels of phosphorylated Akt. Additionally, AKT and mTOR mutations occur relatively rarely in TNBC compared to the hormone-independent HER2+ type [33,40]. Accordingly, given its critical role in tumorigenesis, the PAM pathway represents an attractive target for novel therapies. 

**Figure 1:**
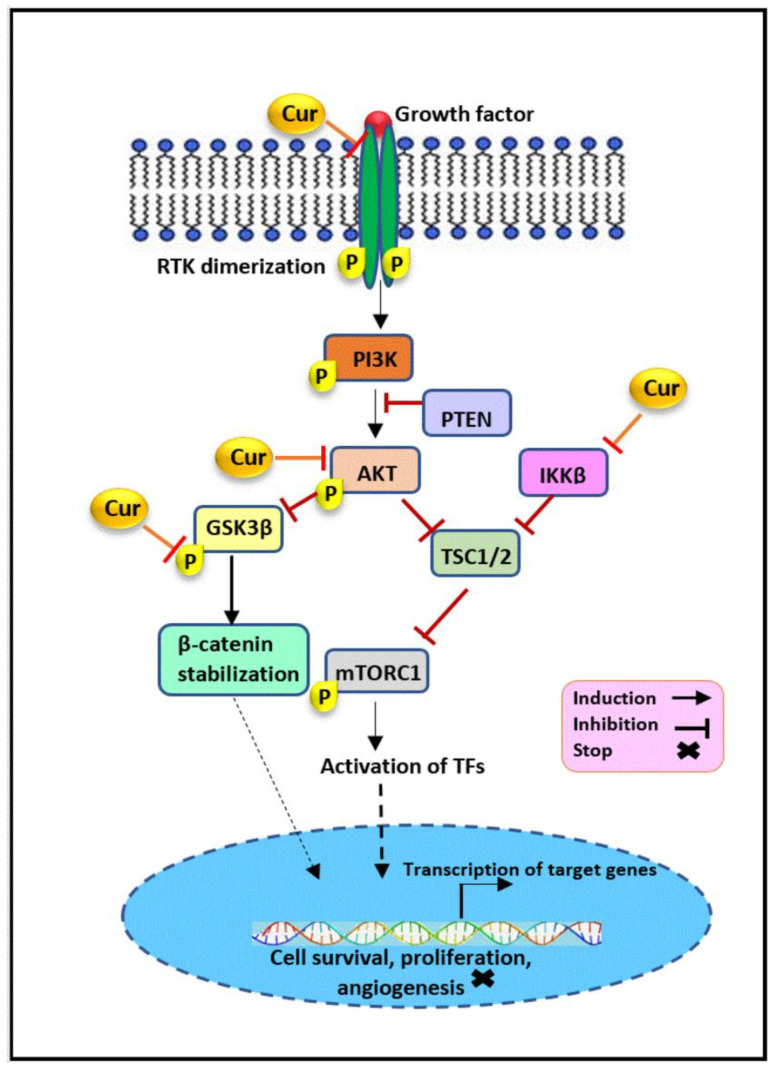
The modulatory effect of curcumin on the PI3K/Akt/mTOR pathway. Curcumin inhibits the PAM signaling pathway through the regulation of its key components. Curcumin downregulates IKKβ, AKT, GSK3β, and HER2 expression, which may facilitate the inhibition of cellular growth, invasion, and metastasis in hormone receptor negative breast cancer. Cur: curcumin, PI3K: phosphatidylinositol-3-kinase, AKT(PKB): protein kinase B, mTORC1: mammalian target of rapamycin complex 1, IKKβ: IκB kinase β, PTEN: phosphatase and tensin homolog, TSC: tuberous sclerosis complex, RTK: receptor tyrosine kinase, GSK3β: glycogen synthase kinase-3β, TFs: transcription factors.

Curcumin has been proven to interfere with the PAM pathway through targeting various signaling molecules, shown in Figure 1, which may facilitate the inhibition of cellular growth, invasion, and metastasis in HR- breast cancer. It has been found that curcumin treatment dramatically downregulated the expression of AKT in a dose- and time-dependent manner in MDA-MB-231 cells, resulting in the suppression of the PAM pathway and, subsequently, the inhibition of cellular proliferation and migrations in TNBC [41]. Curcumin post-translationally regulated Akt protein levels via its degradation. This modification was mediated through the stimulatory effect of curcumin on AMPK activity and the subsequent activation of the autophagy-dependent degradation pathway [41]. Curcumin has the potential to reverse the epithelial-mesenchymal transition (EMT) mechanism by influencing the expression of EMT-related genes in the TNBC cell line. Curcumin treatment downregulated AXL, β-catenin, slug, and vimentin in MDA-MB-231 cells. AXL triggers the PAM pathway through promoting AKT activation, which in turn targets downstream epithelial and mesenchymal regulatory markers leading to tumor cell invasion. Therefore, curcumin may inhibit the invasive ability of TNBC cells through AKT-mediated EMT inhibition [42,43,44]. In addition, it was found that the acquisition of a mesenchymal phenotype in TNBC occurred following doxorubicin treatment is mediated through the PAM pathway. Interestingly, curcumin has been demonstrated to suppress doxorubicin-induced EMT in MDA-MB-231 cells through a reduced expression of p-AKT, p-GSK3β, and β-catenin, and the consequent inhibition of the PAM pathway [45].

In addition, curcumin may have a modulatory effect on IκB kinase β (IKKβ). IKKβ is an upstream kinase that plays a significant role in regulating the PAM pathway through its association with mTORC1 in MDA-MB-453. It binds directly to TSC1, a repressor of mTORC1, and, via its phosphorylation, disrupt the TSC1/TSC2 complex function and subsequently enhances mTORC1 activity, contributing to tumorigenesis. Given IKKβ’s role in hormone-independent HER2+ breast cancer and evidence showing the potential of the curcumin as a IKKβ inhibitor, curcumin may exert its PAM-mediated effect on MDA-MB-453, at least in part, in this way [46,47]. Another suggested mechanism is the downregulation of oncoprotein NEDD4, as an E3-ubiquitin ligase involved in the post-translational modification of protein like PTEN and its degradation by curcumin. It has been found that the overexpression of NEDD4 is involved in the proliferation and migration of MDA-MB-231 cells. A study on breast cancer tissue has also revealed the importance of elevated NEDD4 expression in promoting breast cancer cell growth, progression, and poor prognosis [48,49]. NEDD4 has been demonstrated to negatively regulate the PTEN protein levels in numerous cancers, such as prostate, bladder, lung, and colon [50,51,52]. However, subsequent studies have shown that there is no such correlation in breast cancer, and NEDD4 promote cancer cell growth by facilitating the activation of Akt. Therefore, it is suggested that curcumin may induce NEDD4-mediated PAM suppression in HR- breast cancer in a PTEN degradation-independent manner, worthy of further investigation [48,53]. Besides, it was noted that curcumin suppressed the basal phosphorylation of Akt in MDA-MB-468 cells and then contributed to the significant inhibition of invasion and proliferation and enabled the occurrence of apoptosis in treated cells [54].

Previously it was also shown that treatment with curcumin reduced the cell viability and migration of SKBR-3 and MDA-MB-231 cells. In the same study, curcumin’s effect on tyrosine kinase was investigated, showing a decrease of HER2 combined with a reduction of Akt phosphorylation in a time- and dose-dependent manner in the SKBR-3 cell line [55]. Similarly, another study has revealed the potential utility of curcumin in downregulating the expression of HER2 mRNA and protein in SKBR-3, representing the inhibition of the HER2-related PAM pathway [56]. Evidence also implies that curcumin treatment led to the time- and dose-dependent inhibition of AKT phosphorylation and suppressed Foxo1 and Foxo3a phosphorylation as its downstream targets in MDA-MB-231 cells [57].

### 2.2. The JAK/STAT3 Pathway

Janus kinase (JAK)/signal transducer and activator of the transcription 3 (STAT3) signaling pathway is a chain of interactions among the proteins within the cell whose role is well characterized in the immune system, cell growth, proliferation, differentiation, cell death, and hematopoiesis. Upon stimulation with various factors, such as epidermal growth factor, interferons and interleukin 6 (IL-6), the corresponding receptors get dimerized. This receptor dimerization promotes the phosphorylation of the JAK protein, their cytosolic domain, and eventually the STAT protein. Two such activated STAT3 proteins become attached with one another and form a STAT3 homodimer, which ultimately translocates into the nucleus and act as the transcription factor via interacting with enhancer or promoter regions of DNA in target genes, resulting in their transcription activation [58,59,60]. A considerable amount of literature from clinical and preclinical studies has extensively revealed the aberrant overactivity of the STAT3 pathway, which plays a vital role in breast cancer progression and development. This role is mediated by its impact on huge numbers of downstream targets involved in proliferation (e.g., cyclin D-1, c-myc), apoptosis (e.g., bcl-2, Bax), metastasis (e.g., MMP-2, -9, Twist), as well as chemoresistance [61,62,63,64,65,66]. Interfering with this oncogenic pathway is thus an attractive therapeutic approach.

**Figure 2:**
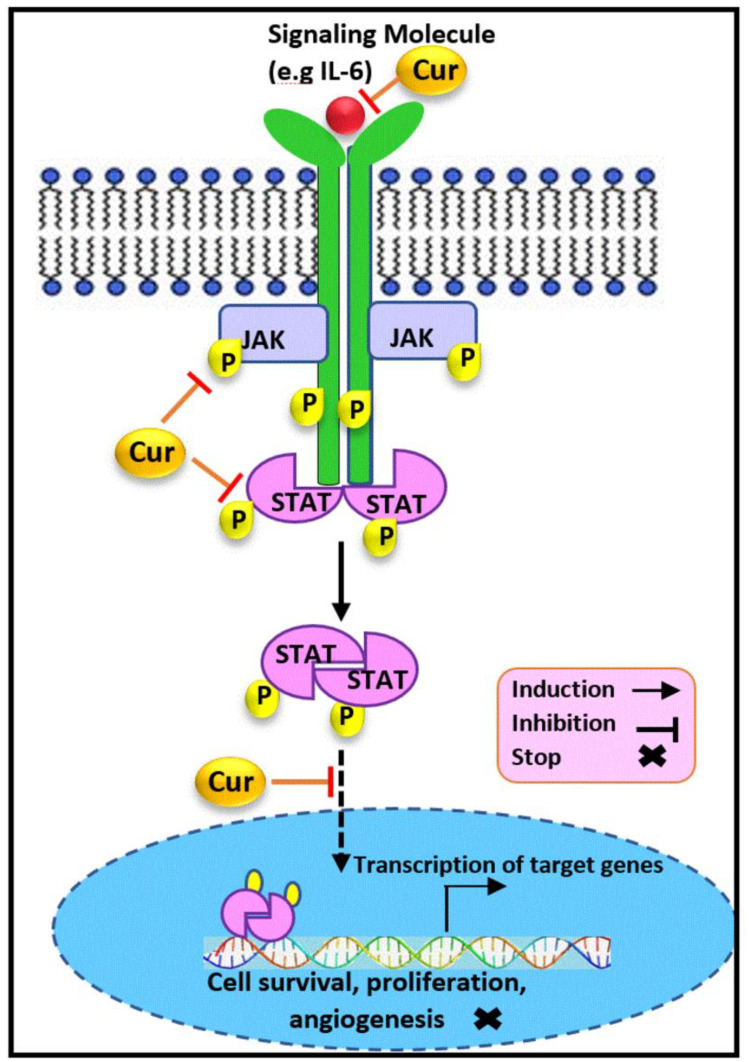
The modulatory effect of curcumin on the JAK/STAT3 pathway. Curcumin inhibits the JAK/STAT3 signaling pathway through the regulation of its key components. Curcumin downregulates STAT, JAK, and IL-6 expression and inhibits STAT translocation into the nucleus, which results in the suppression of cell proliferation, invasion, and metastasis in hormone receptor negative breast cancer. Cur: curcumin, JAK: janus kinase, STAT: signal transducer and activator of transcription, IL-6: interleukin-6.

The JAK/STAT3 signaling pathway has been demonstrated as another target of curcumin, as illustrated in Figure 2. Considerable evidence demonstrates that STAT3 is upregulated and constitutively activated in approximately 70% of breast tumors, which are mostly TNBC. The aberrant activity of STAT3 is linked to survival, stem cells self-renewal, immune evasion, angiogenesis and metastasis, multidrug resistance, and other functions in TNBC cells [67,68,69]. Moreover, suppressing the STAT3-mediated metabolism may inhibit the breast cancer cellular proliferation [70,71,72]. Furthermore, STAT3 expressions have been indicated to be correlated with HER2 amplification. Importantly, a HER2-STAT3 signaling pathway has been determined in hormone-independent HER2+ breast cancer stem cell as well [73,74,75]. Therefore, the blockade of STAT3 by inhibitors is considered to be a promising direction for tumorigenesis and metastasis inhibition in breast cancer, and, notably, curcumin has been reported as a potent inhibitor.

Indeed, curcumin treatment exhibited potent growth suppressive activity and repressed the STAT3 phosphorylation in the MDA-MB-231 and SKBR-3. This result was further confirmed through the inhibitory effect of curcumin on the DNA binding capability of STAT3 and its transcriptional activity [76], which were in accordance with previous reports [67,77]. Therefore, curcumin targets STAT3 signaling by blocking STAT3 activation in vitro. Besides, 3-dimensional culture conditions of TNBC cells revealed that curcumin prevented the tumor-sphere formation and invasiveness of MDA-MB-231 cells [78]. The findings suggested that curcumin induced the inhibition of STAT3 phosphorylation and therefore inhibited its translocation into the nucleus, which resulted in a slightly reduced NFĸB-STAT3 protein interaction and downregulation of CD44 as the marker of cancer stem cell phenotype. Hence, curcumin blocked STAT3-mediated signaling, which contributed to the suppression of the cancer stem cell phenotype in TNBC [78].

A further study also showed that curcumin treatment potentially reduced the active STAT3 expression, which led to a downregulation of its downstream targets and, subsequently, to the suppression of cellular proliferation, colony formation, and cell migration, along with apoptosis induction in treated MDA-MB-231 breast cancer cells [79]. Likewise, it is obvious that breast cancer cells are co-present together with normal mammary cells. Interestingly, it has also been reported that curcumin has the potency to inhibit the paracrine signaling stimulatory effects from TNBC, MDA-MB-231 cells, on non-cancerous mammary epithelial, MCF-10A cells, mediated by blocking the Stat3 phosphorylation [80]. A recent study conducted on wide type (Wt) and forced growth hormone (GH) expressing MDA-MB-453 and MDA-MB-231 cells has demonstrated that curcumin exposure not only reduced the cell viability and exerted its anti-invasive and metastatic effect, but also caused a dose-dependent reduction in GH expression in GH-expressing breast cancer cells. Additionally, an enhanced concentration of curcumin also overcame drug resistance in those cell lines [81]. Interestingly, the findings revealed that curcumin exposure attenuated protein expression and inhibited the phosphorylation of JAK2, STAT3, STAT5, and STAT1 in Wt and GH+ breast cancer cells. Concomitantly, the diminished expression of the wide range of their downstream targets, such as Ras, c-fos, c-raf, c-jun, c-myc, vimentin, snail, and ß-catenin, has also been reported to contribute to the potent impact of curcumin on the respective treated cells [81]. Therefore, curcumin has been found to modulate GH-induced aggressiveness and suppress the JAK/STAT signaling mechanism activation in triple negative and hormone-independent HER2+ breast cancer cells. 

Besides, the persistent autocrine expression of IL-6 cytokine has been considered as an important contributor to progression, resistance, and immune suppression in TNBC. IL-6 also plays a critical role in transforming the dormant breast cancer cells into the actively growing tumor [82,83]. On the other hand, IL-6 activation has been reported to trigger the STAT3 phosphorylation and its activity [84,85,86]. It has been previously shown that the production of IL-6 was significantly reduced in TNBC in vivo 4T1 following curcumin treatment [83]. Hence, another mechanism of curcumin’s modulatory effect on the STAT3 pathway has been suggested to be its impact on IL-6, which is able to turn on the initiation of the IL-6/JAK/STAT3 pathway.

### 2.3. ERK/MAPK Pathway

The mitogen-activated protein kinase (MAPK)/extracellular signal-regulated kinase (ERK) pathway, also known as the Ras-Raf-MEK-ERK pathway, is a transduction cascade transmitting an extracellular signal from mitogens like EGF into the series of signaling events which ultimately promotes cell division, cell proliferation, survival, and cell differentiation. The binding of the mitogen to the cell-surface RTK leads to its dimerization and phosphorylation, and to the recruitment of Grb2/Sos. As a consequence, Ras is activated, and the sequential phosphorylation and activation of Raf, MEK, ERK1 and ERK2 occur. Ultimately, phosphorylated ERK translocate to the nucleus and regulate the transcription of a variety of genes involved in stimulating growth and proliferation [87,88,89]. Besides, c-Jun N-terminal kinas (JNK) and P38MAPK, also known as stress-responsive MAPKs, are the other two MAP kinase pathways functioning in humans which are involved in inflammation, cell growth, and differentiation, as well as apoptosis [87,90]. An altered MAPK signaling pathway plays a vital role in breast cancer progression and development. MAP kinases are significantly correlated with invasion, metastasis, chemoresistance, and poor prognosis in triple negative and hormone-independent HER2 +breast cancer [91,92,93,94]. Thereby, the kinase components of these MAPK pathways have been investigated as putative targets by kinase inhibitors for breast cancer therapy.

Transforming growth factor beta 1(TGF-β1) has been found to be highly associated with cancer invasion and metastasis in late-stage breast cancer. In addition to TGF-β/Smad signaling pathway, TGF-β1 can also trigger tumor growth and regulate cell migration and invasion via Smad-independent mechanisms through the MAP kinase pathway activation [95,96,97]. It was reported that nontoxic doses of curcumin exposure (≤10 μM) resulted in the suppression of ERK1/2 and p38MAPK phosphorylation, stimulated by TGF-β1 in a concentration- and time-dependent manner in MDA-MB-231 cells. Besides, the downregulation of TGF-β2 expression was also found to follow curcumin treatment. Therefore, curcumin exerts its anti-invasion and migratory effect through the inhibition of TGF-β1/MAPK pathway-mediated cell migration [98]. 

Furthermore, the overexpression of EGF, a potent mitogen, and its related receptor, EGFR, is a common feature in breast cancer, regulating the MAPK activity pathway. Enhanced EGFR expression is involved in the progression of tumors to hormone independence [99]. In this regard, curcumin was found to inhibit the phosphorylation of EGFR and ERK1/2 triggered by EGF, and to suppress the ERK activity in the MDA-MB-468 cells [54]. A significant inhibition of JNK, another MAPK, has also been reported following curcumin treatment, correlated with the inhibition of its upstream activator, MKK4. Subsequently, the same study revealed the downregulation of the level of nuclear c-fos and c-jun expressions as the downstream target of the ERK and JNK pathways, in MDA-MB-468 cells [54]. Another study also reported the lower expression levels of phosphorylated ERK1/2 and EGFR in curcumin-treated MDA-MB-468 cells. The findings revealed that curcumin prevented cellular proliferation and triggered apoptosis occurrence mediated by the inhibition of the EGFR/ERK pathway in TNBC cells [29]. Moreover, the incubation of the MDA-MB-231 cells with curcumin led to the reorganization of the fatty acid profile of the breast cancer cell membrane, accompanied by the modulation of p-P44/42 MAPK, ERK1/2, and EGFR expression. An increase in the stearic acid level along with the reduction of arachidonic acids, omega-6 linoleic, and omega-3 have been reported as the membrane remodeling in TNBC cells following curcumin treatment [100].

**Figure 3:**
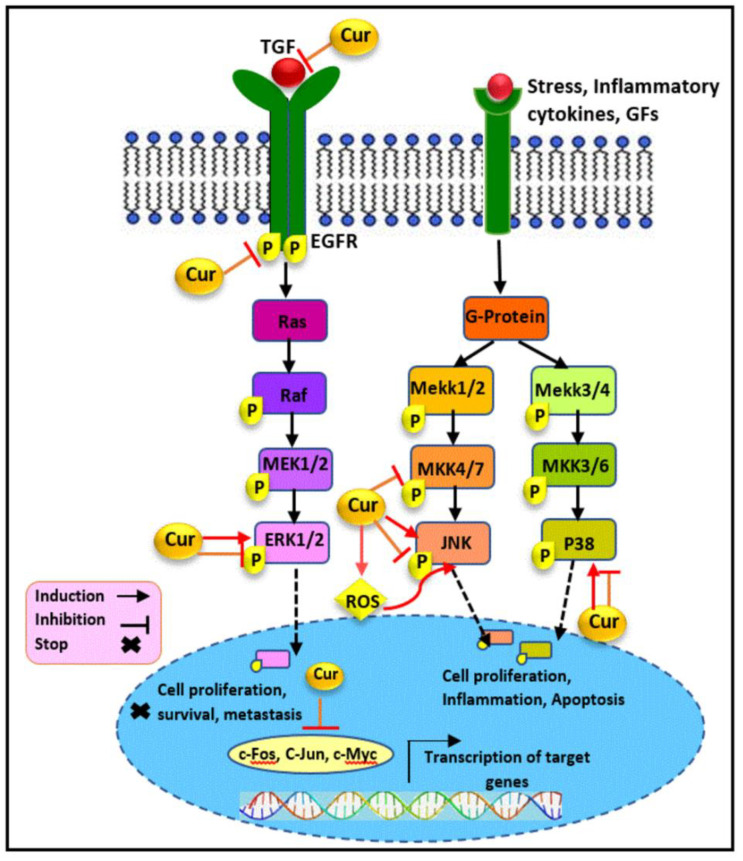
The modulatory effect of curcumin on the MAPK pathway. Curcumin exerts its anticancer activity through the modulation of key components involved in the MAPK signaling pathway. Curcumin targets TGF, EGFR, ERK1/2, MKK4/7, JNK, and P38 molecules and downregulates the expression level of nuclear c-Myc, c-Fos, and c-Jun as the downstream targets of the MAPK pathway, which results in the inhibition of cellular proliferation and migration as well as apoptosis induction in hormone receptor negative breast cancer. Cur: curcumin, G-protein: guanine nucleotide-binding protein, TGF: transforming growth factor, GFs: growth factors, EGFR: epidermal growth factor receptor, Raf: rapidly accelerated fibrosarcoma, MAPK: mitogen-activated protein kinase, ERK: extracellular signal-regulated kinase, MEK: MAPK/ERK kinase, Mekk: Mek kinase, MKK: MAPK kinase, JNK: c-Jun N-terminal kinases.

As shown in Figure 3, MAPK signaling pathways not only promote cellular proliferation and survival, but can also mediate cell death depending upon the cell types and stimuli [101,102]. In this regard, several lines of evidence illustrated that curcumin, under certain circumstances, induces cancer cell death through MAPK activation. The published literature has demonstrated that the upregulation of the enhancer of zeste homolog 2 (EZH2), involved in cell cycle regulation, is correlated with tumor invasiveness and aggressive clinical behavior as well as the poor prognosis of breast cancers [103]. It was found that curcumin diminished EZH2 expression via the phosphorylation and activation of ERK, JNK, and p38 in MDA-MB-435 cells [104]. Similarly, curcumin-mediated apoptosis and autophagy have been detected through the increased activity of ERK, JNK, and Beclin1 in treated MDA-MB-231 [105]. The subsequent study on MDA-MB-231 and Hs578T has demonstrated that curcumin suppresses their proliferation, migratory potential, and induced cell cycle arrest and apoptotic cell death. The immunohistochemistry of clinical tissue specimens demonstrated the curcumin-induced attenuation of ki-67 and proliferation-associated nuclear antigen (PCNA), in the respective tumor tissues. The findings also represented an increase in phosphorylated JNK expression and, subsequently JNK pathway activation, which was associated with enhanced ROS generation. In agreement with analysis, curcumin was also found to prevent tumor growth and metastasis in a MDA-MB-231 xenograft mouse model. Therefore, the anticancer activity of curcumin is meditated via the ROS/JNK signaling pathway [106]. Although reports revealed that the growth inhibition of SKBR-3 cells induced by curcumin treatment was not associated with the suppression of MAPK P38 and ERK expression or phosphorylation [107,108], another study demonstrated that curcumin treatment on SKBR-3 cells resulted in the inhibition of ERK phosphorylation and was also able to sensitize hormone-independent HER2+ cells to TNF-related apoptosis inducing ligand (TRAIL) [109]. Furthermore, the increased phosphorylation of JNK found in curcumin-treated SKBR-3 cells led to the induction of JNK-dependent apoptosis [108].

### 2.4. NF-κB Pathway

**Figure 4:**
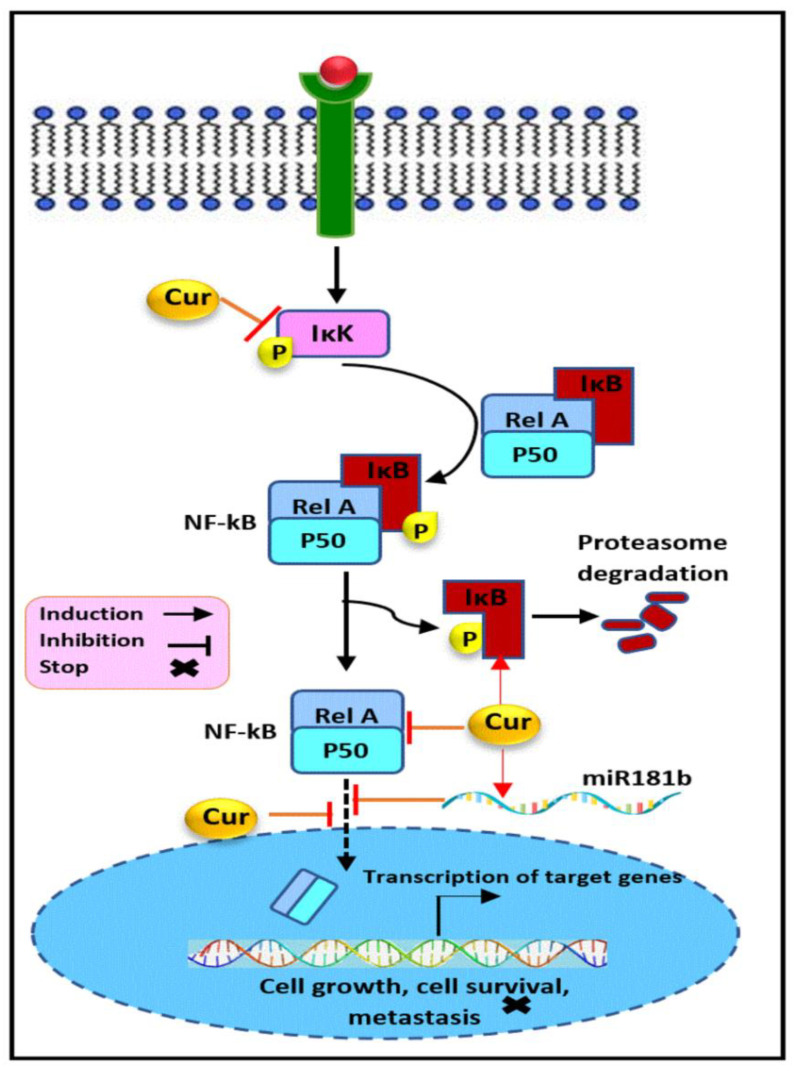
The modulatory effect of curcumin on the NF-κB pathway. Curcumin inhibits the NF-κB signaling pathway through the regulation of its key components. Curcumin upregulates IκB and miR181b expression and downregulates NF-κB and IKK, and suppresses the NF-κB translocation into the nucleus, which results in the inhibition of cellular proliferation, survival, metastasis, and angiogenesis in hormone receptor negative breast cancer. Cur: curcumin, IκK: inhibitor of kappa B kinase, IκB: inhibitor of NF-κB, Rel A: p65.

The nuclear factor-kappa B (NF-κB) pathway plays a critical role in promoting cell survival, inflammation, differentiation, and cell growth [110]. As shown in Figure 4, it is stimulated upon the binding of the signaling molecules, e.g., TNF-α or cytokines, to their corresponding receptors at the surface of the cell membrane, leading to the receptor conformational changes and the subsequent recruitment, phosphorylation, and activation of the inhibitor of kappa B kinase (IKK). NF-κB is a heterodimer complex protein, e.g., RelA (p65) and p50, rather than a single protein, presenting an inactive form associated with a group of inhibitory proteins known as inhibitors of NF-κB (IκB) in cytosol. Other members of the NF-κB transcriptional factor family are NF-κB1 (p50), NF-κB2 (p52), RelB, and c-Rel. Activated IKK, in turn, leads to the phosphorylation of IκB, which results in provoking IκB polyubiquitination and its subsequent proteasome-mediated degradation. This causes NF-κB dissociation and its translocation to the nucleus, where it binds, as the transcriptional factor, as dimer in enhancers and promoters of downstream targets such as pro-inflammatory, anti-apoptotic, and cytokine-related genes to regulate their transcription, and hence numerous critical cellular process. Besides, other receptors of the TNFR superfamily such as LTβR, BAFFR, TNFR2, CD40L, and receptor tyrosine kinases such as EGFR and the AKT protein may also stimulate the activation of the NF-κB pathway in addition to the canonical activation [110,111,112].

There is increasing evidence that the NF-κB pathway is constitutively activated and is a frequent characteristic in TNBC and hormone-independent HER2+ subtypes mediated through overexpression of EGFR, HER2, and NF-κB subunits including p50, c-Rel, and p65. Its aberrant activation has been strongly implicated in breast cancer pathogenesis and prognosis and facilitates the development of hormone-independent invasiveness and metastasis, angiogenesis, as well as chemo- and radio-resistance [113,114,115,116]. This evidence cooperatively highlights the urgency and significance of targeting the NF-κB pathway for breast cancer prevention and therapy.

Curcumin was found to induce the inhibitory action on NF-κB activation in SUM159, and MDA-MB-231, accompanied by a decrease in its target expression, including IAP-1 and surviving as anti-apoptotic factors. These findings show that the anti-invasion ability of curcumin might be mediated through MDA-9/Syntenin-mediated NF-κB regulation in treated TNBC cells [112]. Another study demonstrated the inhibitory activity of curcumin through the suppression of NF-κB p50 gene expression in 4T1 TNBC cells [117]. Besides, the attenuation of nuclear and cytoplasmic NF-κB p65 protein expression and the subsequent modulation of the NF-κB-inducing genes, including cyclin D1, CDK4 and MMP-1, P21, were indicated as the mechanism basis for the anti-proliferative and anti-invasive properties of curcumin against MDA-MB-231 [118].

Besides, several lines of evidence have identified curcumin as a potent chemosensitizer through the regulation of the NF-κB pathway. It was shown that curcumin suppressed paclitaxel-activated NF-κB in in MDA-MB-435. This effect was mediated through the hindrance of IKK activation, its phosphorylation, and its degradation. The same study also revealed the apoptosis induction and decline in expression of anti-apoptotic (XIAP, IAP-1, IAP-2, Bcl-2, and Bcl-xL), proliferative (COX-2, c-Myc, and cyclin D1), and metastatic proteins (VEGF, MMP-9, and ICAM-1) in response to curcumin treatment. In support of these observations, an in vivo experiment confirmed the role of the curcumin -mediated NF-κB inhibition in preventing breast cancer metastasis in a xenograft mice model [119]. The other study also revealed the beneficial role of curcumin in decreasing the chemoresistance to gemcitabine through the blockage of NF-κB activity, and therefore the enhancement of their anticancer effect against MDA-MB-231 cells [120]. Similarly, in vitro and in vivo results obtained from a former study indicated that the sensitization of curcumin-treated breast cancer cells to paclitaxel and cyclophosphamide was mediated through the inhibition of NF-κB (p50 and p65), protein kinase C (PKC), histone deacetylase (HDAC), and telomerase activity in MDA-MB-231 cells and a breast cancer mouse model [121]. In line with these results, curcumin potentiates 5-fluorouracil-triggered toxicity in MDA-MB-231 cells through the thymidylate synthase-dependent downregulation of NF-κB, IKK phosphorylation, and IkBα degradation [122].

Another study provides evidence that curcumin reduces the expression of the p65 subunit of NF-κB in TNBC cells accompanied by the marked downregulation of FABP5, PPARβ/δ, and eventually VEGF-A and PDK1 as the downstream target genes involved in cell proliferation, survival, and angiogenesis. The findings also revealed curcumin’s capability to reverse the resistance of MDA-MB-231 and MD-MB-468 to retinoic acid and inhibit cancer cell growth by targeting and suppressing the NF-κB-mediated FABP5/PPARβ/δ pathway [123]. It has been reported earlier that curcumin blocked the lipopolysaccharide-induced EMT procedures through the modulation of the expression of motility and invasiveness marker, e.g., vimentin and E-cadherin, in MDA-MB-231 breast cancer cells. These effects were triggered through the downregulation of NF-κB p65 and the consequent repression of snail protein, a direct downstream transcription factor. Curcumin reversed cellular EMT characteristics through the inhibition of NF-κB-Snail signaling activation [124]. Likewise, another report has also demonstrated that a mild curcumin treatment exerts a disruptive impact on the adhesion and rolling behavior of SKBR-3, MDA-MB-231, and MDA-MB-468 cells and decreases the metastatic potential of circulating tumor cells most likely via acting on the NF-κB pathway [125]. An in vivo TNBC mouse model also indicated the inhibitory role of curcumin on tumor growth and angiogenesis. In breast tumor tissues of the curcumin-treated group, a significant suppression of NF-κB p65 and a deregulation of NF-κB-related genes expression, cyclin D1, and PECAM-1 were detected [126]. 

Furthermore, the anti-metastatic effects of curcumin in MDA-MB-231 breast cancer cells was correlated with the reduction of inflammatory cytokines CXCL1 and CXCL2 mRNAs and proteins, which are both tightly related to metastases. The underlying mechanism involved the regulation of NF-κB p65 and IkBa expression in treated breast cancer cells [127]. In line with these findings, mechanistic studies conducted on MDA-MB-231 cells and several primary human breast cancers have revealed the miR181b upregulation involved in the curcumin-induced, downregulatory effect on CXCL1 and -2, which mediated its anti-metastatic potential [128]. miR181b is also a potent regulator of NF-kB signaling by targeting importin-α3, essential for the translocation of NF-κB to the nucleus and NF-κB inhibition [129]. Besides, the modulation of miR181b triggered by curcumin has a functional impact on tumor cell proliferation, invasion, and apoptosis. Additionally, the upregulation of miR181b in metastatic breast cancer cells was found to suppress metastasis development in an in vivo mice model [128].

### 2.5. P53 Pathway

P53 is a tumor suppressor and transcription regulator forming a homo-tetramer to induce the transcription of almost 500 target genes that are responsible for various cellular mechanisms, mainly DNA repair and cell cycle arrest, as well as apoptotic cell death. P53 is activated in response to diverse forms of stimuli such as hypoxia, DNA damage, and oncogene activation ultraviolet light. It is known as the genome guardian due to protecting and maintaining the genome’s integrity and stability via triggering severely DNA-damaged cells to death. Under normal conditions, p53 is a short-lived protein where p53 expression is precisely controlled through an autoregulatory feedback loop in which murine double minute 2 (MDM2), as the negative regulator, destabilizes p53 [130,131,132].

P53 may promote apoptosis through a transcription-independent and -dependent mechanism which is regulated by diverse environmental factors, signals, and cell types. Within the nucleus, the transcription-dependent mechanism is mediated through the interaction of p53 with basal transcriptional machinery components and the enhancement of the expression of genes such as Bax, Noxa, and Puma. However, in the mitochondria, its non-transcriptional modes of action are chiefly mediated through its molecular interaction with anti- (Bcl-2 and Bcl-XL) and pro-apoptotic (Bak and Bax) members of the Bcl-2 family. Therefore, any loss in p53 function may contribute to tumor growth and cancer development correlated with a deficiency in the cell cycle checkpoint, instability of genome, cellular immortalization, and irregular cell survival and proliferation [132,133].

The TP53 gene, a tumor suppressor, is the most common mutated gene in breast cancer and has been reported to be more frequently altered in hormone-independent HER2+ (72%) and TNBC (80%) compared to the luminal A (12%) and B (29%) subtypes of breast cancer. There is significant evidence implying that mutated nonfunctional p53 is involved in tumorigenesis and progression and also associated with worse clinical outcomes, low survival rate, prognosis, and chemoresistance in breast cancer patients [134,135,136,137]. Therefore, anticancer agents with an ability to reactivate or boost the p53 pathway have been considered as promising drug candidates in breast cancer therapy.

**Figure 5:**
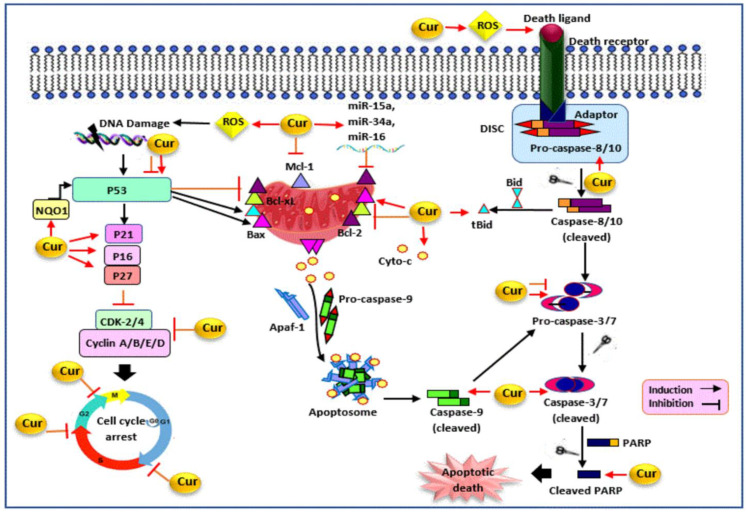
The modulatory effect of curcumin on p53, cell cycle, and apoptosis pathways. Curcumin inhibits cell cycle progression and induces apoptosis by targeting various molecules. Curcumin triggers ROS generation and alters the expression level of caspases (caspase-3/7, 8, 9), PARP, Bcl-2 family proteins (Mcl-1, Bcl-xL, Bid, Bax, Bcl-2), cytochrome-c, and miRNAs (miR-34a, miR-15a, miR-16), which results in apoptotic cell death in hormone receptor negative breast cancer. Curcumin also modulates P53 expression, upregulates CDKI (P21, P27, P16), and downregulates cyclins (cyclin A, B, E, D) and CDKs (CDK-2, -4), which results in cell cycle arrest in hormone receptor negative breast cancer. Cur: curcumin, DISC: death-inducing signaling complex, Bcl-2: B-cell lymphoma 2, Bid: BH3 interacting-domain death agonist, tBid: truncated Bid, Bax: Bcl-2 associated X protein, Bcl-xL: B-cell lymphoma-extra large, Mcl-1: myeloid cell leukemia 1, APAF-1: apoptotic protease activating factor-1, Cyto-c: cytochrome c, PARP: poly (ADP-ribose) polymerase, ROS: reactive oxygen species, CDK: cyclin-dependent kinase, NQO1: NAD(P)H dehydrogenase (quinone 1).

The impacts of curcumin as a p53 regulator in hormone-independent breast cancer and the involvement of the fundamental molecular mechanism of this regulation (Figure 5) have been widely investigated. It has been reported that curcumin upregulates p53 expression and regulates MDA-MB-231 proliferation and apoptosis [126,138]. An in vivo study also confirmed the upregulation of p53 and the reduction of Ki67 protein levels as the underlying mechanism of suppression of breast tumor growth in curcumin-treated mice groups [139]. Moreover, curcumin treatment upregulated p53 expression and induced caspase-dependent apoptosis in SKBR-3 and MDA-MB-231. These effects were accompanied by the upregulation of Bax and Bid and the downregulation of Bcl-2 and Bcl-xL in treated cells [140,141,142]. An additional study reported that the ROS-triggered DNA damage in curcumin-treated MDA-MB-231 cells resulted in p38-MAPK-mediated p53 expression. These effects were accompanied by the regulation of Bax, Bcl-2, p16, and p21 expression, which eventually led to apoptosis induction and cell cycle arrest [143]. In contrast, it has been reported that curcumin treatment decreases the expression level of p53 and phosphorylated p53 (S392, S15, S392) in MDA-MB-231, SKBR3 and EMT6 cells, representing the induction of p53- independent apoptosis in treated cells [108,144,145,146,147]. Moreover, it has been documented that Notch1 overexpression is correlated with a highly expressed mutant p53 (R280K) in MDA-MB-231 cells, in which mutant p53 acts as its transcriptional activator. This study on TNBC cells inferred that curcumin treatment led to apoptosis induction accompanied by a declined expression of mutant p53, Notch1, and Hes1 as its downstream target. The researchers concluded that the occurrence of cellular apoptosis was modulated by the inhibition of the mutant p53-Notch1 signaling axis following the exposure of the cancer cells to curcumin [148,149]. Likewise, another study also indicated curcumin-induced apoptosis. Additionally, the significance of curcumin in the p53 stability regulation of MDA-MB-231 cells was determined where 20 µM curcumin treatment enhances the half-life of the P53 protein in TNBC cells. This effect was also demonstrated, along with the increased level of NQO1, which might play an important role in p53 stabilization [150].

### 2.6. Wnt/β-Catenin Signaling Pathway

Signaling by the secreted glycolipoprotein factors of the Wnt family via β-catenin, as the transcription co-activator, regulates multiple developmental processes during embryogenesis and adult homeostasis through its critical role in cell growth, differentiation, and cellular metabolism [151,152]. Once the Wnt signaling molecule presents itself, it binds to a Frizzled receptor and triggers the activation of the co-receptor LRP, leading to a transfer of the biological signal to a Disheveled (Dvl) protein within the cytoplasm. The activated Dvl protein then prevents the hydrolysis of β-catenin by a destructive multiprotein complex composed of several proteins, such as GSK3, APC, Axin, and CK1. As a result, β-catenin remains stable inside the cytosol. Accordingly, β-catenin, as the core component of this pathway, accumulates, migrates to the nucleus, and displaces the transcriptional repressor Groucho protein through the formation of the β-catenin/TCF/LEF transcriptional complex. Eventually, the transcription of Wnt-responsive genes such as slug, cyclin D1, VEGF, c-myc, and MMPs will be initiated. However, in the absence of extracellular Wnts, the β-catenin destruction complex promotes the phosphorylation, ubiquitinylation, and degradation of β-catenin in proteasome. Therefore, this pathway is maintained in an off state [153,154].

The aberrant Wnt/β-catenin signaling network, through altered functions or levels of its components, is a key driver of breast cancer progression, phenotype shaping, and recurrence [153,155]. Dysregulated Wnt/β-catenin signaling, as a characteristic of TNBC, correlated with the tumorigenesis, TNBC stem cell pluripotency, clinicopathological parameters, poor clinical outcomes, and therapeutic resistance, as well as brain and lung metastases [154,156,157,158,159]. Increasing evidence has also implicated the role of the overactivity of the Wnt/β-catenin pathway in the progression, promoting an EMT-like phenotype and drug resistance in hormone-independent HER2 + breast cancer [160,161,162]. Given its importance in the transcription of various target genes supporting cell proliferation and metastasis, the therapeutic inhibition of the Wnt/β-catenin signaling cascade has a significant role in the management of breast malignancy.

**Figure 6:**
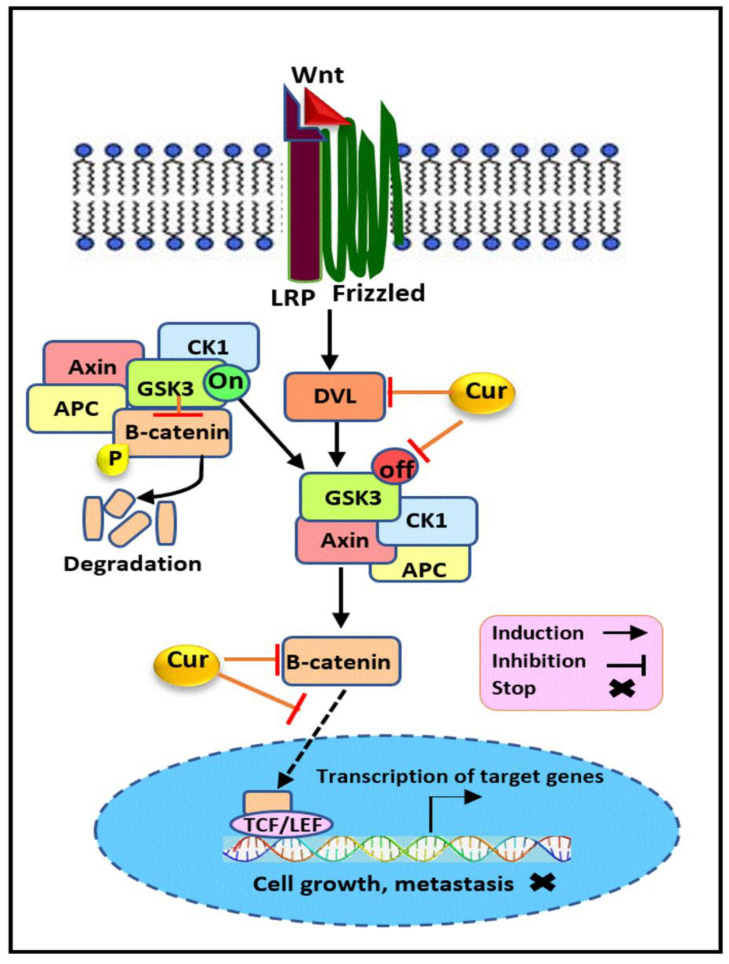
The modulatory effect of curcumin on the Wnt/β-catenin pathway. Curcumin inhibits the Wnt/β-catenin signaling pathway through the regulation of its key elements. Curcumin downregulates β-catenin, Dvl, and the inactive form of GSK3 and inhibits β-catenin translocation into the nucleus, which results in the suppression of cellular proliferation and metastasis in hormone receptor negative breast cancer. Cur: curcumin, Wnt: wingless/integrated, Dvl: disheveled, LRP: lipoprotein receptor-related protein, GSK3: glycogen synthase kinase-3, APC: adenomatous polyposis coli, CK1: Casein kinase 1, TCF/LEF: T cell factor/lymphoid enhancer factor.

Some evidence relates to curcumin’s potential on the modulation of the Wnt/β-catenin signaling pathway (Figure 6) in breast cancer. It has been previously reported that the growth suppressive impact induced by curcumin in MDA-MB-231 cells is mediated via its regulatory effect on this pathway. A Western blot analysis of treated cells with 20µM curcumin demonstrated the multiple suppressive effects on its components, including the marked downregulation in the expression levels of β-catenin, Dvl, cyclin D1, and slug proteins. However, the lack of a significant alteration in the expression of the E-cadherin and GSK3β protein was also reported upon exposure of TNBC cells to curcumin [163]. Besides, an immunofluorescence analysis has also illustrated the modification of the sub-cellular localization of Wnt/β-catenin pathway elements. Accordingly, these alterations included a significant decline in the cytoplasmic and nuclear expression level of the Dvl protein, along with a marked reduction in the nuclear level of β-catenin, cyclin D1, and slug in treated TNBC cells. Additionally, the G2/M cell cycle arrest and the enhanced expression of cytokeratin 18 (CK18), as an early event during apoptosis, showed the anti-proliferative activity and apoptosis occurrence, respectively, following curcumin treatment. These finding suggest that curcumin exert its anticancer effect on TNBC cells through the abrogation of Wnt/β catenin signaling mediated via the modulation of its key elements [163]. Existing evidence strongly implies that stem cell markers have been regulated as the downstream target of β-catenin [164,165,166]. Curcumin’s vital role in the modulation of metastases and cancer stem cell activity has been shown to be mediated through the Wnt/β-catenin pathway inhibition. The study conducted on MDA-MB-231 and its derived breast cancer stem cells (BCSC) revealed that the anti-metastatic effect of curcumin was induced through the regulation of EMT-related markers, including β-catenin, vimentin, E-cadherin, N-cadherin, and fibronectin. The suppression of stem cell-like characteristics via the downregulation of Sox2, Oct4, and Nanog was also detected in treated TNBC cells [167]. Similar results have also been obtained with curcumin against SUM159 BCSCs. The results showed that curcumin suppressed GSK3β phosphorylation, in its inactive form, leading to the reduced expression of β-catenin and its downstream target c-myc in treated BCSCs. In line with these results, the downregulation of stem cell markers including CD44, Nanog, ALDH1A1, and Oct4 was also observed. Besides, the suppression of the sonic hedgehog pathway due to the downregulatory effect of curcumin on shh, Smo, Gli1, and Gli2 contributed to BCSC inhibition [168]. Another report also indicated that curcumin exerts its anti-invasive properties on MDA-MB-231 cells through the downregulation of EMT-related genes such as β-catenin, N-cadherin, vimentin, and AXL, representing its capability to hinder the EMT process [43].

### 2.7. Apoptosis

Apoptosis is a highly regulated process of programmed cell death, with a critical role in the normal tissue homeostasis and development. It also occurs as a defense mechanism to eliminate potentially cancerous, damaged, and virus-infected cells [169,170]. Apoptosis machinery is conducted through two distinct signaling pathways. A mitochondria-mediated (intrinsic) pathway triggered by hypoxia, oxidative stress, and DNA damage involves the release of cytochrome-c from the mitochondrial intermembrane space into cytosol, followed by apoptosome complex formation and caspase-9 activation. Anti- and pro-apoptotic members of the Bcl-2 family also regulate the permeabilization of the mitochondrial outer membrane [171,172]. However, the death receptor-mediated (extrinsic) pathway is stimulated by the interaction of extracellular death ligands such as TNF-α, FAS, and TRAIL, with their corresponding death receptors of the TNF receptor superfamily. This receptor-ligand binding triggers DISC complex formation and subsequently caspase-8/10 proteolytic activation. Both pathways eventually activate caspase-3 and 7, executing cell death [172,173]. In addition to these two conventional pathways, the NF-κB, MAPK, PI3k/AKT, STAT3, and β-Catenin pathways may also induce apoptosis [174,175,176,177,178,179,180]. 

In addition to its physiological significance, the deregulation of apoptosis is critically involved in the pathogenesis of various diseases, ranging from cancer to neurodegenerative disorders. It is well established that the evasion of apoptosis may promote tumor initiation, progression, metastasis, and resistance to therapy in breast cancer [172,181,182]. Reduced apoptosis or its resistance in cancer cells can be mediated through numerous mechanisms. For instance, the overexpression of the pro-survival Bcl-2 protein is common in TNBC breast cancer. Besides, Bcl-2 is upregulated in approximately 50% of hormone-independent HER2+ breast cancers, which makes it a clinical prognostic marker in breast cancer [183,184,185,186,187]. There is also a close relationship between dysregulated caspase expression and the development of hormone-independent breast cancer which is also involved in its clinicopathological features and poor overall survival [188,189]. Altered death receptor signaling also contributes to apoptosis resistance in breast cancer [190,191,192]. Collectively, given the critical role of apoptosis evasion in promoting the pathogenesis and progression of breast cancer tumors, therapeutic targeting of the apoptotic machinery in cancer cells holds great promise in the anticancer drug discovery and development.

Curcumin has been proven to promote cellular apoptosis by altering the expression of various cellular molecules, as shown in Figure 5. Poly (ADP-ribose) polymerase (PARP) is implicated in DNA repair, cell survival, transcriptional regulation, and apoptosis. The cleavage of PRAP into its fragments causes its enzymatic role deactivation, which eventually leads to cell death [193,194]. It was shown that curcumin increased cleaved PARP, cleaved caspase-3, cleaved caspase-7, cleaved caspase-9, caspase-3, caspase-8, cytochrome-c, Bax, and Bid expression and decreased Bcl-2, Mcl-1, and Bcl-xL expression, which resulted in apoptosis induction in MDA-MB-231 cells and tumor growth inhibition in an in vivo xenograft model [142,195,196,197]. Besides, the downregulation of caspase-3 expression was also observed in curcumin-treated MDA-MB-231 [198]. Moreover, curcumin-induced apoptosis was accompanied by increased caspase-3 and PARP cleavage, Bax upregulation, and surviving downregulation in SKBR-3, MDA-MB-231/HER2 cells, MDA-MB-468, and HCC1806 [108,199,200].

Another mechanism of curcumin-induced apoptotic cell death is through altering the expression of miRNAs. Curcumin upregulated the expression of miR-15a and miR-16 in SKBR-3 cells, which resulted in decreased Bcl-2 expression and apoptosis occurrence [201]. In another study, curcumin suppressed the proliferation, invasion, and induced apoptosis via the miR181b upregulation and subsequent CXCL-1 and -2 downregulation, as the pro-metastatic and inflammatory cytokines, in MDA-MB-231 cells [128]. A further study also revealed that curcumin-induced miR-34a expression led to the downregulation of Bcl-2 and Bmi-1 proteins in MDA-MB-231 and MDA-MB-435 [202]. In addition, curcumin-induced apoptosis is also linked to its ability to trigger ROS generation. It was shown that curcumin upregulated the polyamine catabolic enzyme expressions, PAO and SSAT, which resulted in ROS induction and the subsequent activation of intrinsic and extrinsic apoptotic pathways in wide-type and growth-hormone-expressing MDA-MB-453 and MDA-MB-231 cells [81]. Besides, the involvement of ROS induction by curcumin in mitochondrial dysfunction increased cleaved PARP and caspase-3, and the Bax/Bcl-2 expression ratio has been determined in MDA-MB-231 and SKBR-3 cells [56,203].

Furthermore, fatty acid synthase (FAS) is a key metabolic enzyme that is highly expressed in breast cancer and is therefore a putative tumor target. It was shown that curcumin treatment inhibited the cell growth and induced apoptosis dose-dependently, via the inhibition of FAS in MDA-MB-231 cells. This inhibition was associated with the regulation of AKT phosphorylation, Bax, and Bcl-2 protein expressions [204]. FAS inhibition–mediated apoptosis was also reported in curcumin-treated SKBR-3 cells [205]. Moreover, Tafazzin (TAZ) and Yes-associated protein (YAP) are the key effectors of the Hippo signaling pathway. The upregulation of TAZ and YAP is involved in cellular proliferation, EMT, apoptosis suppression, and therapeutic resistance in breast cancer [206,207]. In a recent study, it was shown that curcumin downregulated TAZ and YAP expression in MDA-MB-231 cells and tumor xenografts in mice. These alterations inhibit the proliferation, migration, invasion, apoptosis induction, and tumor growth suppression in TNBC models [208]. In addition, ion channels play a significant role in tumorigenesis due to their function in proliferation, apoptosis, and metastasis [209,210,211,212]. A recent research has shown that curcumin regulated the expression levels of potassium and non-potassium ion channel genes, which resulted in the upregulation of cleaved caspase-3, cytochrome c, and haem oxygenase-1, along with the downregulation of cIAP-1, claspin, and survivin protein in MDA-MB-231 cells [147]. Curcumin-mediated apoptosis could also be linked to its potential to induce DNA damage and increase the expression level of H2AFX, PARP1, BRCA1, and RAD51 in treated MDA-MB-231, HCC1937, MDA-MB-468, and HCC1806 cells [200,213].

Epigenetically mediated apoptosis is another underlying mechanism of curcumin. The expression level of the enhancer of zeste homolog 2 (EZH2), deleted in liver cancer 1 (DLC1), is negatively correlated in breast cancer, where the upregulation of EZH2 and the downregulation of DLC1 have been reported in breast cancer tissue and MDA-MB-231 cells. It was found that curcumin restored DLC1 expression by inhibiting EZH2 expression, which led to growth inhibition and apoptosis induction in MDA-MB-231 cells and in an in vivo xenograft model [214]. Additionally, Ras-association domain family 1 isoform A (RASSF1A) is a potential tumor suppressor correlated with the modulator of apoptosis 1 (MOAP-1), which promotes Bax conformational changes and its activation. It was found that curcumin suppressed cell proliferation and induced apoptosis via the upregulation of the RASSF1A, Bax, and caspase-3 protein expression in MDA-MB-231 and MDA-MB-468 cells [215,216,217].

### 2.8. Cell Cycle

Cell cycle is a highly regulated process of cell duplication which involves several checkpoints to ensure its proper progression. Multiple cyclins and cyclin-dependent kinases (CDK) form cyclin-CDK complexes which determine a cell progression through the cycle. However, when the cells no longer divide, cyclins are degraded and, subsequently, the deactivation of CDKs and cell cycle arrest occur [218,219,220]. Besides, CDK inhibitors (CDKIs) comprised of Ink4 (inhibitor of CDK-4) including p15, p16, p18, and p19 and kinase inhibitor protein (Kip) including p21, p27, and p57 as well as retinoblastoma (RB1) protein negatively regulate the cell cycle progression. [219,221,222].

The cell cycle machinery was deregulated at multiple levels in breast cancer cells, promoting cancer development and resistance to therapy [219,223,224]. Cyclin D1 is probably the most extensively studied cyclin in breast tumors, and its overexpression has been reported in more than 50% of breast cancer cases, as detected in TNBC and hormone-independent HER2+ [224,225]. Cyclin D1 also has CDK independent functions through its binding to histone acetylases, histone deacetylases, and nuclear receptors to regulate cell proliferation, growth, and differentiation. Moreover, it functions in the DNA repair system by binding to RAD51 involved in the homologous recombination of DNA during double strand break repair [225,226,227,228]. TNBC also display a frequent alteration of RB1 and the DNA damage response gene, BRCA1. Besides, overexpression of the CDK4 is also a common feature among breast cancer types, with the highest frequency in the hormone-independent HER2+ subtype [224,229]. Thereby, due to their vital role in breast cancer cell proliferation, the cell cycle regulatory proteins are the potential targets in cancer therapy. 

Curcumin has been shown to inhibit the cell cycle progression in various phases and alter the expression of different cell cycle proteins in TNBC and hormone-independent HER2+ breast cancer (Figure 5). It has been previously reported that curcumin treatment caused an increase in the accumulation of cells in the S and G2/M phases in MDA-MB-468 cells and exerted its dual impact on cancer cell progression [54]. It was also found that a 10 µM concentration of curcumin led to G2/M phase arrest, accompanied by the upregulation of p21 expression and the downregulation of cyclin A, B1, D1, and E expression in SKBR-3 cells [56]. In addition, the induction of G0/G1 cell cycle arrest was associated with p21 and p27 upregulation and cyclin D1 downregulation in SKBR-3 cells [108]. Another report revealed that the curcumin-mediated EZH2 downregulation is correlated with the G1 arrest in MDA-MB-435 cells [104]. Furthermore, a recent study has shown that curcumin inhibited the expression of cyclin D and cyclin E in SKBR-3 and MDA-MB-231 cells [230]. Besides, curcumin upregulated p21, p16, and p53 and downregulated cyclin E, cyclin D1, CDK-2, and CDK-4 in MDA-MB-231 cells. These effects were mediated via ROS induction and p38-MAPK activity, which led to G1/S and G2/M arrest and cell death in treated cells [143,197]. In addition, the downregulation of STAT3-mediated cyclin D1 and c-Myc following curcumin treatment resulted in G1 arrest in MDA-MB-231 [142]. An increased p21 and p53 expression along with a reduced Rb protein expression also caused G2/M cell cycle arrest in MDA-MB-231 and MDA-MB-453 cells [81,106]. The reports also revealed the upregulation of p27 and p21 expression and the downregulation of cyclin E, CDK-2, and CDK-4 via curcumin-mediated SKP2 inhibition in MDA-MB-231 and MDA-MB-231/HER2 cells [57,199]. Additionally, the downregulation of PCNA, RAD50, RAD51, Nbs1, BRCA1, BRCA2, and Mre11, involved in the DNA damage response and repair, was observed in curcumin-treated MDA-MB-231 cells [143,198,231]. Curcumin also regulated the cellular localization of BRCA1 by triggering its cytoplasmic retention in MDA-MB-468 and HCC1806, having functional BRCA1, but not in BRCA1-defective HCC1937 [200].

## 3. Clinical Trial

A large number of reported preclinical studies of in vitro and in vivo models support the promising role of curcumin as a potential chemopreventative and chemotherapeutic agent in breast cancer treatment. Accordingly, the clinical research trial of curcumin and its synergistic effect with other chemotherapeutic drugs in enhancing breast cancer therapy have been evidenced. In a phase I clinical trial conducted in 2010 in 14 patients with advanced and metastatic breast cancer, the feasibility and tolerability of curcumin, docetaxel chemotherapy, as a microtubule inhibitor, and their combination were explored. In this trial, a daily oral dose of 0.5 g of curcumin was given and further escalated until a dose-limiting toxicity occurred, along with intravenous docetaxel (100 mg/m^2^) [232]. Based on the obtained findings, no enhanced incidence of hematological toxicity was observed. However, the tested combination significantly decreased the VEGF levels. Although it was found that a daily administration of 8 g curcumin is the maximum tolerable dose, the phase II dose of 6 g/day, 7 days, every 3 weeks, in combination with a standard dose of docetaxel, has been reported to be suitable for further evaluation [232]. Moreover, due to the low bioavailability of curcumin through oral administration, a phase II clinical trial of 150 women investigated the effectiveness and safety of treatment with intravenous curcumin, compared to placebo, in combination with paclitaxel chemotherapy among patients with metastatic and advanced breast cancer [233]. In this trial, the patients received intravenously either paclitaxel (80 mg/m^2^)-placebo or paclitaxel-curcumin (300 mg solution) combinations once a week for 12 weeks. The findings demonstrated that, with respect to the objective response rate and physical performance, curcumin in combination with paclitaxel had a superior impact on patients compared to the placebo after 12 weeks of treatment and a short-term follow-up. Besides, an adverse effect analysis suggested not only the lack of safety concerns of intravenous curcumin but also its clinical efficacy on the reduction of fatigue, an extreme feeling of tiredness, or a lack of energy as the most common side effects of chemotherapy [233].

Besides, in a phase II study, 30 breast cancer patients undergoing radiotherapy following completion of their chemotherapy have been analyzed in order to investigate the curcumin potential versus placebo to reduce the DNA binding of NF-κB and the activation of its downstream target. In this study, curcumin as Meriva (500 mg BID), which is a curcumin formulation improving its absorption, has been orally given to the respective patients. The researchers propose that, by decreasing the activity of NF-κB and ultimately plasma IL-6, fatigue may improve in breast cancer patients taking Meriva [230]. Currently, a phase I clinical trial study is recruiting 20 participants in order to assess the effect of the oral administration of curcumin on apoptosis- and cell proliferation-related biological changes in primary tumors of invasive breast cancer patients (stages I, II, or III) [234].

## 4. Conclusions

**Figure 7:**
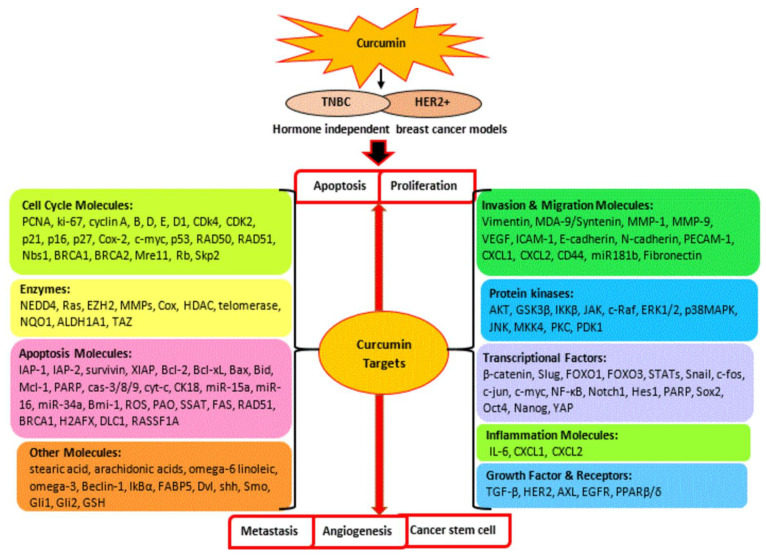
Multi-target action of curcumin against TNBC and hormone-independent HER2 positive breast cancer. Curcumin induces apoptotic cell death and suppresses cellular growth, proliferation, metastasis, angiogenesis, and cancer stem cell through the regulation of multiple molecular targets.

Overall, the molecular basis of hormone-independent breast cancer is correlated with the dysregulation of various key signaling cascades consisting of the PI3K/Akt/mTOR pathway, JAK/STAT pathway, MAPK pathway, NF-ĸB pathway, p53 pathway, Wnt/β-catenin, and apoptosis and cell cycle pathways. The current review deepens and expands our knowledge of anti-breast cancer activity of curcumin by interfering with these oncogenic signaling pathways, which leads to the regulation of cell survival and proliferation, metastasis, angiogenesis, cancer stem cell, and cell death in TNBC and hormone-independent HER2+ breast cancer. Curcumin has been shown to interact with multiple molecular targets including various kinases, transcription and growth factors, receptors, apoptosis, and cell cycle regulatory molecules, etc., to exert its therapeutic role in hormone-independent breast cancer, as shown in Figure 7. Therefore, a detailed understanding of its multifunctional anticancer action may provide a framework for future studies and insights to improve its efficiency in clinical practice. Moreover, curcumin has enhanced the effectiveness of the chemotherapeutic drugs paclitaxel, gemcitabine, doxorubicin, 5-fluorouracil, and docetaxel and overcome drug resistance in hormone-independent breast cancer, warranting the further exploration of various curcumin-therapeutic agents’ synergism in a larger scale of pre-clinical and clinical studies. In clinical studies, its application either alone or in combination has also revealed its significant role in improving breast cancer therapy and reducing adverse effects such as fatigue and radiation-induced dermatitis in patients. Additionally, the development of a novel curcumin formulation and a more efficient delivery system continues to be a subject of great interest for overcoming its poor bioavailability, which limits its clinical applications.
